# The use of HEAVEN criteria to predict difficult laryngeal view and intubation failure with direct and video laryngoscopy

**DOI:** 10.1186/s13049-019-0654-y

**Published:** 2019-08-13

**Authors:** Liu-Jia-Zi Shao, Shao-Hua Liu, Fu-Shan Xue

**Affiliations:** Department of Anesthesiology, Beijing Friendship Hospital, Capital Medical University, NO. 95 Yong-An Road, Xi-Cheng District, Beijing 100050 People’s Republic of China

**To the Editor**

With great interest we read the article written by Nausheen et al. [1] recently published in the Scandinavian Journal of Trauma, Resuscitation and Emergency Medicine. By using the multiple logistic regression analyses, they showed that in patients receiving emergency rapid sequence intubation with direct laryngoscopy (DL) and video laryngoscopy (VL), each component of the HEAVEN criteria (Hypoxaemia, Extremes of size, Anatomic challenges, vomit/blood/fluid, Exsanguination, Neck mobility) and the total number of the HEAVEN criteria were significantly associated with both difficult laryngoscopy (Cormack-Lehane grade III/IV) and intubation failure at the first attempt with and without oxygen desaturation. However, we have some questions about the paper by Nausheen et al. and would invite the authors to comment on these issues.

First, it is usually considered that no single risk factor can provide a reliable prediction for a difficult laryngoscopy and intubation failure, as each risk factor individually has a rather low positive predictive value [2]. If more risk factors of a difficult airway are found in the same patient at the same time, however, the likelihood of a difficult laryngoscopy and intubation failure will increase [3]. Very strangely, in their table 2, we noted that the odds ratios of the total number of the HEAVEN criteria for intubation failure using DL and VL at the first attempt with and without oxygen desaturation were significantly lower than those of a single anatomic challenge component of the HEAVEN criteria. This indicates that compared with a single anatomic challenge component, the total number of the HEAVEN criteria cannot provide an improved prediction for intubation failure using DL and VL at the first attempt. Thus, we question the predictive reliability of the total number of the HEAVEN criteria for intubation failure at the first attempt during emergency rapid sequence intubation with DL and VL.

Second, only providing the odds ratios of the HEAVEN criteria for a difficult laryngoscopy and intubation failure with DL and VL at the first attempt by the multiple logistic regression analyses is incomplete to determine its predictive ability. We suggest that both the sensitivity analysis and the receiver operating characteristic curve analysis should further be performed to obtain the sensitivity, specificity, positive, and negative predictive values of the HEAVEN criteria for a difficult laryngoscopy and intubation failure with DL and VL in the validation and development sets, as performed in previous studies [4, 5]. By providing the predicted probabilities and observed frequencies for a difficult laryngoscopy and intubation failure at the first attempt based on each component of the HEAVEN criteria and the total number of the HEAVEN criteria, the readers can estimate whether there is a good overall agreement between predicted probabilities and observed frequencies in the validation and development sets. Furthermore, the area under the receiver operating characteristic curve can also indicate the discrimination ability of the HEAVEN criteria in predicting a difficult laryngoscopy and intubation failure at the first attempt.

## Data Availability

Data sharing not applicable to this article as no datasets were generated or analyzed during the current work.
